# Ceftizoxime loaded ZnO/l-cysteine based an advanced nanocarrier drug for growth inhibition of *Salmonella typhimurium*

**DOI:** 10.1038/s41598-021-95195-0

**Published:** 2021-07-30

**Authors:** M. S. Bacchu, M. R. Ali, M. A. A. Setu, S. Akter, M. Z. H. Khan

**Affiliations:** 1 Department of Chemical Engineering, Jashore University of Science and Technology, Jashore, 7408 Bangladesh; 2 Laboratory of Nano-Bio and Advanced Materials Engineering (NAME), Jashore University of Science and Technology, Jashore, 7408 Bangladesh; 3 Department of Microbiology, Jashore University of Science and Technology, Jashore, 7408 Bangladesh

**Keywords:** Biochemistry, Biotechnology, Chemical biology, Health care, Chemistry

## Abstract

l-Cysteine coated zinc oxide (ZnO) nano hollow spheres were prepared as a potent drug delivery agent to eradicate *Salmonella enterica* serovar Typhimurium (*S. typhimurium*). The ZnO nano hollow spheres were synthesized by following the environmentally-friendly trisodium citrate assisted method and l-cysteine (L-Cys) conjugate with its surface. ZnO/L-Cys@CFX nanocarrier drug has been fabricated by incorporating ceftizoxime with L-Cys coated ZnO nano hollow spheres and characterized using different techniques such as scanning electron microscope (SEM), attenuated total reflection Fourier transform infrared (ATR-FTIR), and X-ray diffraction (XRD) etc. Furthermore, the drug-loading and encapsulation efficiency at different pH levels was measured using UV–vis spectrometer and optimized. A control and gradual manner of pH-sensitive release profile was found after investigating the release profile of CFX from the carrier drug. The antibacterial activity of ZnO/L-Cys@CFX and CFX were evaluated through the agar disc diffusion method and the broth dilution method, which indicate the antibacterial properties of antibiotics enhance after conjugating. Surprisingly, the ZnO/L-Cys@CFX exhibits a minimum inhibitory concentration (MIC) of 5 µg/ml against *S. typhimurium* is lower than CFX (20 µg/ml) itself. These results indicate the nanocarrier can reduce the amount of CFX dosed to eradicate *S. typhimurium*.

## Introduction

Antibiotic is the greatest invention at last century used to eradicate pathogens by reducing pathogen growth and destroying them^1,2^. Conventional antibiotics have drawbacks, such as multidrug-resistance (MDR) bacteria, minimum bioavailability, less diffusion capacity to the outer membrane, etc.^3^. World health organization (WHO) defines these MDR bacteria as microorganisms that can grow in the presence of one or more antibiotics^4^. The recovery from these MDR strains caused the healthcare-associated infection to become a more significant challenge day by day. High doses of antibiotics are administrated to overcome this challenge, which may generate toxic and adverse effects. A promising strategy to manage MDR bacteria-caused infections is applying nanoparticles (NPs) to restrain the MDR microorganism^5–7^. NPs can be the carrier of antibiotics and enhance the antibacterial activity of the antibiotics^8^. This nanocarrier can enter cell's intracellular endocytic pathways and deliver the drug at a targeted position^9^. The NP-based carrier for antibiotics significantly reduces the antibiotic doses required to eradicate pathogen several times than the dose of the free antibiotics.

*Salmonella typhimurium* is a highly gram-negative pathogenic bacteria which causes salmonellosis for human and animals^10^. The global concern about the treatment of *S. typhimurium* poses infection is day by day increasing due to its antibiotic resistance behavior. The antibiotic susceptibility properties of *S. typhimurium* were decreased, and this pathogen is resistant to chloramphenicol, tetracycline, streptomycin, ampicillin, etc.^11^. For this reason, we choose ceftizoxime (CFX) (member of cephalosporin antibiotic group) to treat *S. typhimurium* caused the infection. CFX is a third-generation antibiotic which is effective for both gram-positive and gram-negative bacteria. The minimum amount of CFX requires to inhibit pathogenic growth for human body tissue causes different adverse effects^12^. It is crucial to improve the therapeutic efficiency of CFX, which can be achieved by developing CFX loaded nano-drug carrier (NDC) with the minimum side effect^13^. This NDC increases the therapeutic efficiency and reduces the drug consumption rate by the sustainable release of antibiotics. Different inorganic nanoparticles are widely used as carriers for antibiotics such as gold nanoparticles, silver nanoparticles, iron oxide nanoparticles, silica nanoparticles, zinc oxide nanoparticles, etc. Different biocompatible polymers are generally used in NDC for coating with nanoparticles to achieve specific targeted and sustainable release^14^. We choose a noble NDC based on l-cysteine (L-Cys) coated hollow Zinc oxide nanosphere to reduce antibiotic consumption. ZnO is a safe metal oxide approved by the US food and drug administration (FDA), suggesting the micro to larger size ZnO are generally safe, but nano shape might be toxic. It is accepted that discrete polymer coating on the surface of ZnO reduces its toxicity and makes it more efficient for drug delivery^15^. Nano-shaped ZnO exhibited antibacterial properties over different pathogenic bacteria such as *Escherichia coli*, *Staphylococcus aureus*, etc.^16–22^. A variety of nanostructured ZnO is generally used in the drug delivery system, such as quantum dots, nanospheres, nanoshells, nanosheets, nanorods, nano hollow spheres, nanobelts, and nanobelts, etc. Among them, nano hollow spheres take greater attention due to substantial internal space in their core. This core helps in drug transport, specific targeted drug delivery to the particular regime, and maintains release profile^23,24^.

l-Cysteine (L-Cys) is a member of amino acid groups that are widely used in targeted drug delivery because of its active groups (carboxylic –COOH, amines –NH_2_, and thiol -SH). The thiol group present in L-Cys gives some exciting properties such as metal conjugating, damage skin repairing, and cross-linking^25^. The three functional groups of L-Cys may interact with the ZnO sphere surface where the thiol group of L-Cys reacts with Zn^2+^ of ZnO and form a complex^26^. L-Cys works as a scavenger free radical that create oxidative stress to cause cellular damage^27^.

In this work, we present CFX loaded NDC named ZnO/L-Cys@CFX for controlled drug delivery for the growth inhibition of *S. typhimurium*. Here, the carrier matrix is L-Cys coated ZnO nano hollow sphere synthesized in simple and environmentally-friendly trisodium citrate assisted method. Finally, the antimicrobial test, such as the zone inhibition analysis and minimum inhibitory concentration (MIC) analysis, is carried out over *S. typhimurium* to characterize the NDC.

## Materials and methods

### Materials and reagent

All chemicals and reagents used in this study were in high analytical grade. Trisodium citrate (Na_3_C_6_H_5_O_7_), Zinc nitrate hexahydrate (Zn(NO_3_)_2_‧6H_2_O), sodium chloride (NaCl) were purchased from Merck (Germany). Hexamine (C_6_H_12_N_4_) was brought from PT. SMART LAB (Indonesia). CFX and L-Cys were purchased from Aladdin Reagent Ltd. (China). Salmonella Shigella (SS) agar and Mueller Hinton agar were purchased from Oxoid(UK). Ultrapure water from Evoqua (Germany, resistivity > 18 Ω) was used for all types of preparations. Phosphate buffer saline (PBS) of 0.01 M used for the release study was purchased from Aladdin Reagent (China).

### Instruments

Scanning electron microscope (SEM) of different nanoparticles and drug carriers was performed using ZEISS GeminiSEM 500 field emission scanning electron microscope (FE-SEM). A NICOLERT iS20 ATR-FTIR, Bruker D8 Advance PXRD, and Shimadzu lab solution UV–VIS (UV-1900i) were also used to investigate the different physicochemical characterization and drug loading efficiency. An electrochemical workstation of Corrtest CS300 was used to conduct in vitro released behavior of the carrier drug.

### Synthesis of hollow ZnO nanosphere

The ZnO nano hollow spheres were synthesized by following a slightly modified procedure, as previously reported^28^. In short, a 200 ml aqueous solution of 15 mM zinc nitrate hexahydrate, 15 mM hexamine, and 4.08 mM trisodium citrate were transferred into 250 ml round bottom flux made of bomex glass. Then, the flux was sealed using a sealing plug and placed in the oven at 90 °C for two hours. After completing the reaction period, the white color residue was found, and the precipitate was collected by centrifuging at 5000 rpm after naturally cooling. The resulting ZnO was washed with ultrapure water several times to remove the residue and dried at 60 °C for 24 h. The pure hollow shape ZnO was found after annealing at 400 °C for 2 h and finely grinding.

### Preparation of ZnO/L-Cys and ZnO/L-Cys@CFX

To prepare ZnO/L-Cys, 100 mg of hollow ZnO nanosphere, 50 mg of L-Cys were dispersed in 10 ml normal saline (0.85% NaCl) separately. Each solution was ultrasonicated for 5 min and stirred magnetically by using a magnetic stirred for 1 h. After formulating the homogenous solution, the required amount of ZnO and L-Cys solutions were taken in 40 ml normal saline to get a suspension of 1 mg ZnO and 0.5 mg L-Cys. Then, the resulting solution was stirred using a magnetic stirrer for 1 h to prepare L-Cys coated hollow ZnO nanosphere (ZnO/L-Cys).

**Scheme 1:**
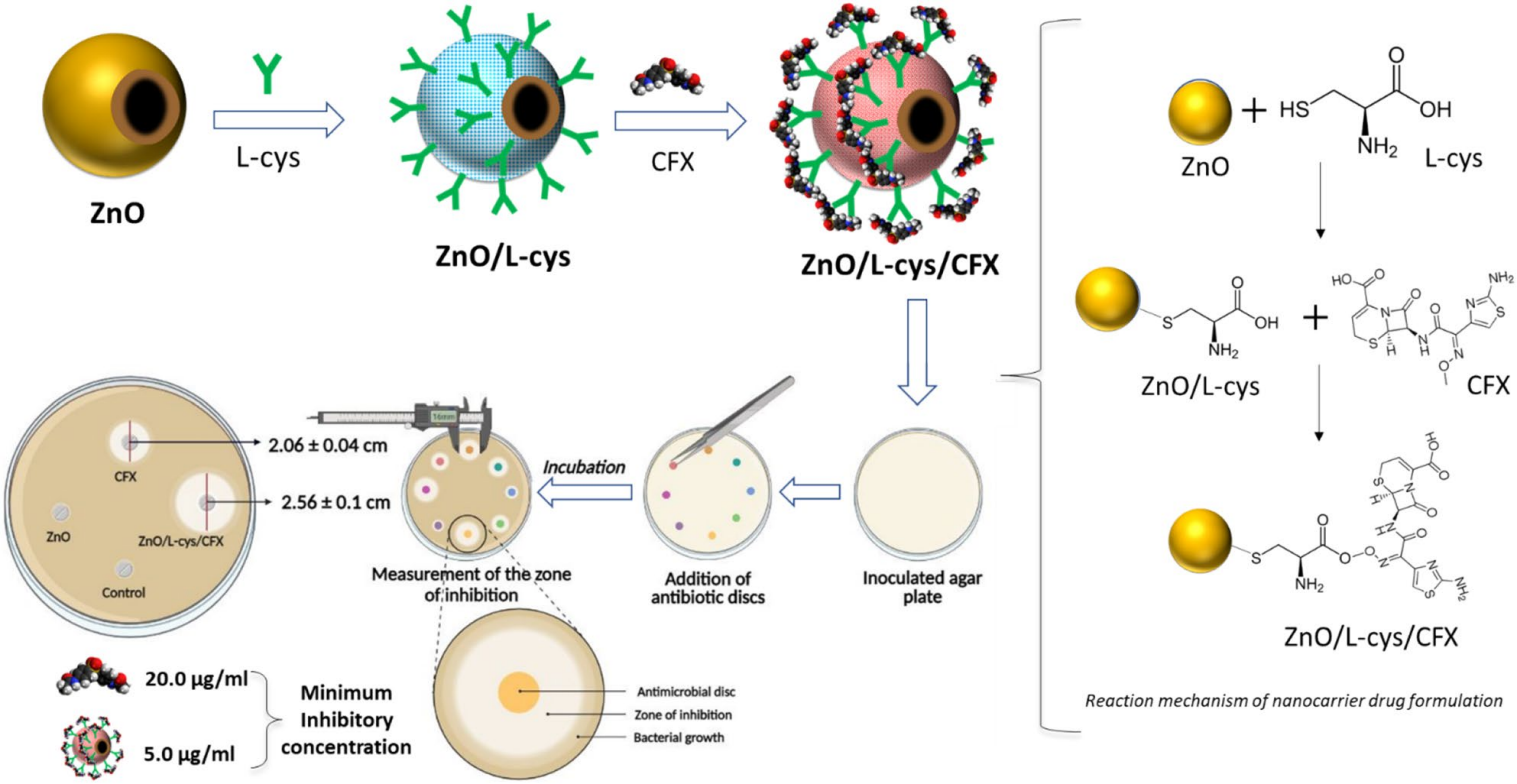
Step-by-step preparation technique of proposed nanocarrier drug and its working principle.

To prepare ZnO/L-Cys@CFX, 1 mg of CFX was immersed in 10 ml normal saline to prepare 100 µg/ml CFX stock solution and stirred magnetically to make a homogenous solution. An aliquot of required volume CFX was added to the ZnO/L-Cys solution to prepare ZnO/L-Cys@CFX where the concentrations of CFX were 1, 5, 10, 20 µg/ml and stirred in a magnetic stirrer for 1 h. The ZnO/L-Cys@CFX of different concentrations (1, 5, 10, 20 µg/ml based on CFX) were denoted as C_1_, C_2_, C_3_, C_4_. Then the prepared solutions were stored at 4 °C and used for different antibacterial tests. Scheme 1 represents the preparation steps of the ZnO/L-Cys@CFX nanocarrier and its reaction mechanism.

### Drug loading and encapsulation efficiency

**Figure 1:**
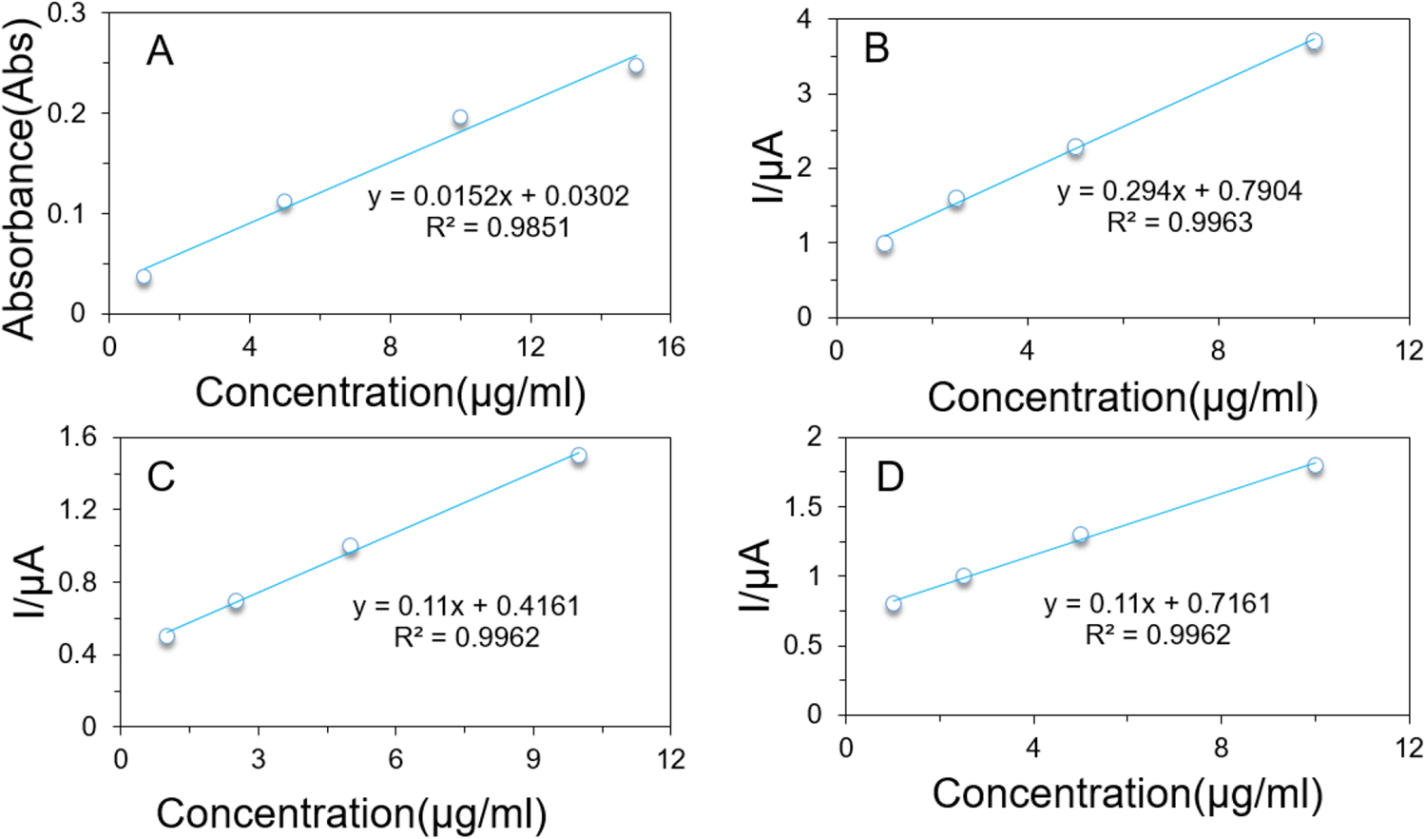
Calibration curve for measuring drug loading efficiency by using UV–Vis spectroscopy (**A**). Calibration curve for evaluating drug release percentage by using the electrochemical sensor at pH 3.5 (**B**), pH 7.4 (**C**), and pH 9.0 (**D**).

The amount of drug loading was determined by using a UV–Vis spectrometer to assess the effect of pH on drug adsorption. For this study, ZnO/L-Cys@CFX (C_3_) was prepared in ultrapure water of different pH values ranging from 1.5 to 7.5 according to a similar procedure as previously stated. Then, the resulting solutions were centrifuged at 6000 rpm, and the supernatant was collected. To determine the amount of drug loading and encapsulation efficiency, the supernatants were analyzed with UV–Vis spectrometer at the wavelength of 298 nm^29^ using a calibration curve of CFX in the concentration of 1–15 µg/ml (y = 0.0152x + 0.0302 and R^2^ = 0.985) shown in Fig. 1A. The amount of drug loading was calculated by subtracting the total amount of drug that remains in supernatants from the total amount of drug initially used. The encapsulation efficiency was calculated by dividing the total amount of drug loading by the total amount of drug loading drug initially used.

### In vitro drug release

For the drug release study, a certain amount of ZnO/L-Cys@CFX was sequentially immersed into 50 ml of PBS solution of (pH 3.5, 7.4 and 9.0) in a water bath at 37 °C with continuous shaking. At specific time intervals, 4 ml of the resulting solutions were withdrawn and the amount of release was calculated by using an electrochemical sensor where MWCNT/P-Cys@MIP modified glassy carbon electrode (GCE) was used as a working electrode, silver/silver chloride (Ag/AgCl) electrode and platinum wire were used as reference and counter electrode. The amount of drug released was evaluated according to our previously reported work^30^. Calibration curves of different pH were also done to determine the amount of CFX released, as shown in Fig. 1B–D.

### Antibacterial tests

#### Bacterial culture

*For the antimicrobial* assay, *we have used* CFX sensitive S. typhimurium *(*ATCC 19585) strain. We resuscitated the lyophilized cells on nutrient broth and culture on MacConkey agar medium. Pure discrete typical colonies from the plate were subcultured on Mullier Hinton agar and used further for the antimicrobial susceptibility tests.

#### Antibiotic susceptibility assay

##### Agar disc diffusion method

We followed the National Committee for Clinical Laboratory Standards (NCCLS) guideline^31,32^ and performed the antibiotic susceptibility assay by modified Kirby–Bauer’s disc diffusion method^33^ on Mueller–Hinton Agar media. We have used CFX antibiotic at different concentrations (1.0. 5.0, 10.0 and 20.0 μg/ml) alone named D_1,_ D_5_, D_10,_ D_20_ and in combination with 1 mg/ml ZnO nano hollow sphere and 0.5 mg/ml l-cysteine cross-linked nanoparticle named C_1_, C_2_,C_3_, and C_4_. The bacterial suspension was prepared using overnight grown pure culture on Muller Hinton agar media into normal physiological saline (0.85% NaCl) and adjusted the turbidity comparing with 0.5% MacFarland standard. A non-absorbent cotton swab was dipped in the suspension and spread over a Muller Hinton agar plate to make a bacterial lawn. Blank antimicrobial susceptibility discs (ThermoScientific Oxoid) of 0.5 cm are placed at the center, and measured antibiotic solutions (with or without nanoparticles) are poured on the disc. The Petri dishes are incubated upside up the position at 37 °C for 18 h. The zone of bacterial growth inhibition around the discs is measured manually using a cm-scale.

##### Broth dilution method to compare the effect of nanoparticles conjugated with antibiotic on bacterial susceptibility

We compare the bactericidal effect of ZnO/L-Cys@CFX and CFX by broth dilution method according to EUCAST, 2003 guidelines of European Society of Clinical Microbiology & Infectious Disease (ESCMID). Ceftizoxime antibiotic concentrations were set at 1.0 μg/ml, 5.0 μg/ml, 10.0 μg/ml and 20.0 μg/ml named D_1_, D_2_, D_3_, D_4_. Tubes with antibiotic alone and, in combination with 1 mg/ml ZnO nano hollow sphere and 0.5 mg/ml l-cystine cross-linked nanoparticle named C_1_, C_2_, C_3_, and C_4_ were prepared in normal saline. All the tubes were inoculated with a measured amount of antibiotic-sensitive S. typhimurium strain. All the tubes were incubated at 37 °C, and after 6 h of an interval, viable bacterial count (CFU/ml) was measured by the drop plate method on MacConkey agar plates. The plates were incubated overnight (17–20 h) at 37 °C. The colony-forming unit (CFU) of the three drops in the countable dilution range were averaged, and the total count was scaled up^34^. The same procedure has been adopted for determining the antimicrobial activity of ZnO nano hollow spheres, L-Cys, and ZnO/L-Cys. In this case, five different concentrations of ZnO nano hollow sphere, L-Cys, and ZnO/L-Cys of three were used as test suspensions and followed the above-mention protocol to detect its effect on the bacterial strain.

## Results and discussions

### Physio-chemical properties of Carrier matrix

**Figure 2:**
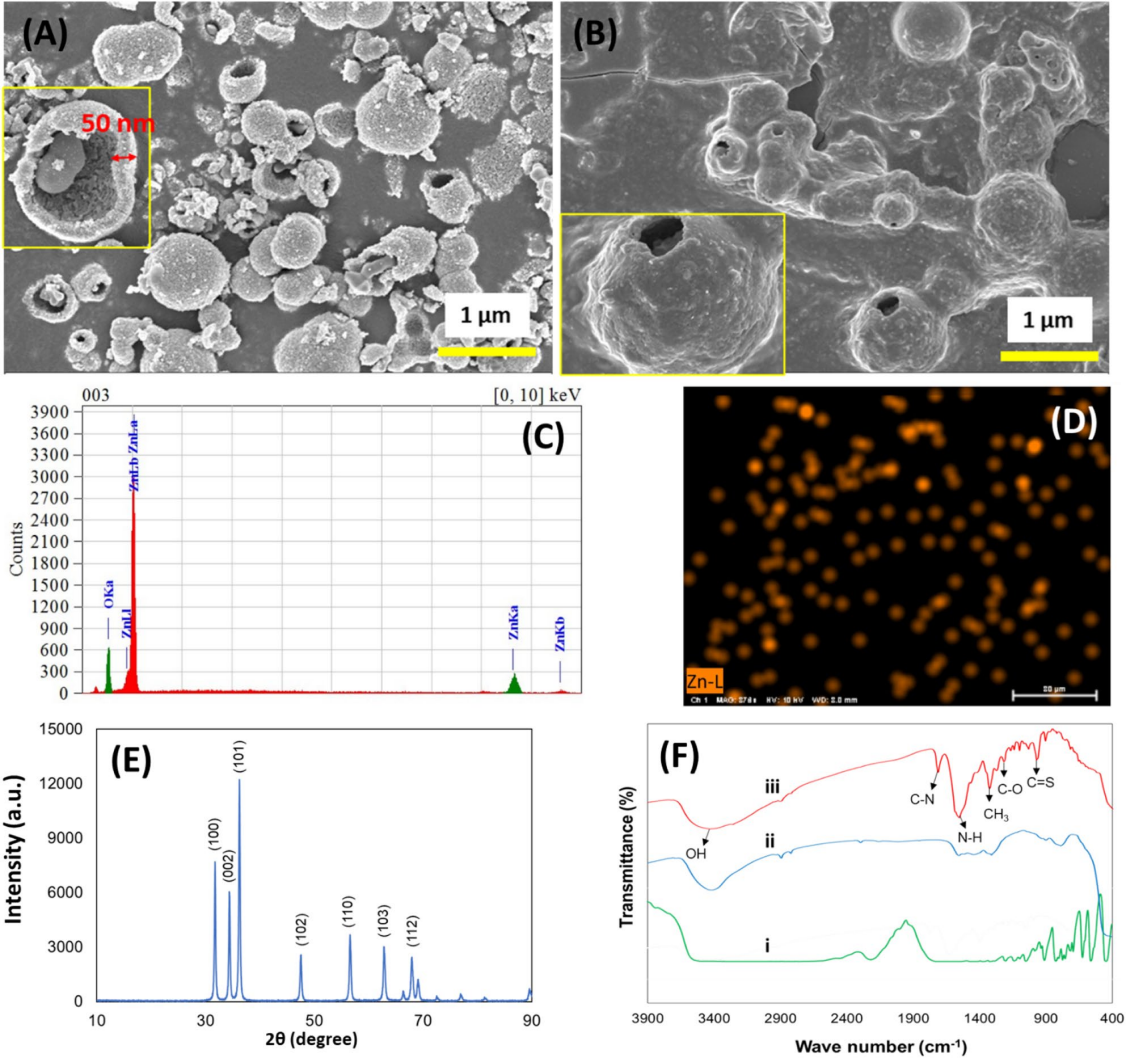
SEM images of as-synthesized ZnO nanosphere (**A**) and ZnO/L-Cys@CFX (**B**). The inset shows a high magnification image of the same. EDX spectra (**C**) and elemental mapping (**D**) of ZnO in the nanocomposite. (**E**) XRD peaks of synthesized pure ZnO sphere and (**F**) ATR-FTIR spectra of (i) L-Cys powder; (ii) ZnO/L-Cys, and (iii) ZnO/L-Cys@CFX.

The morphology of the obtained nanocarrier was characterized using field emission scanning electron microscopy as shown in Fig. 2. Figure 2A shows the low and high magnification SEM images of ZnO hollow spheres. The average diameter of individual particles ranged from 95 to 350 nm with sphere-shaped nanocrystal structures and the thickness of the walls is about 50 nm. The SEM image of the CFX loaded ZnO/L-Cys nanocarrier was shown in Fig. 2B. It can be observed that the overall morphology of the nanospheres remained unchanged even after the incorporation of CFX which suggests strong binding of drug molecules with nanocarrier through cross-linking of L-Cys. The EDX spectra (Fig. 2C) and elemental mapping of the same (Fig. 2D) confirm the presence and uniform distribution of Zn in the carrier drug.

The changes of phase structure and crystallite size of the as-prepared ZnO hollow sphere were investigated by XRD measurement and represented in Fig. 2E. The observed diffraction peaks are in good agreement with the pure ZnO hollow microsphere structure (JCPDS card 36-1451)^35^, which confirm the hollow structure of the sample with P63mc symmetry. No other peaks of impurities and different crystalline phases existed, which ensures the purity of synthesized materials, which is in good agreement with SEM results. The earlier researcher reported the higher entrapment capacity of hollow nanocarriers for greater concentrated release of drugs^14^.

ATR-FTIR analyses were performed to evaluate the functional groups of the nanocarrier surface and the following conjugation of the target drug (Fig. 2F). The presence of a Zn–O stretching mode is confirmed by the intense broadband at 430–500 cm^−1^ in the spectrum of ZnO hollow spheres. The spectrum of L-Cys (Fig. 2Fi) can be labeled as (–NH) bending vibration at 1140 cm^−1^ and while peaks around 900 cm^−1^ prove the –COOH group. In addition, a weak band attributed to the (–SH) group of cysteine molecule is observed near 2350 cm^−1^ which completely disappears (Fig. 2Fii) due to the cleavage of S–H bonds and bonding with ZnO hollow spheres via the thiol group. The bifunctional ligand like L-Cys can afford thiol groups to form a chemical bond on the NPs surface^36^. FTIR spectrums of ceftizoxime loaded ZnO/L-Cys are displayed in Fig. 2Fiii. The spectra revealed the peaks at 3440 cm^−1^ that confirmed the presence of an OH group. The observed other peaks showed changes in recorded phases as the drug was entrapped in the ZnO/L-Cys structure.

### Effect of pH on drug loading and efficiency of encapsulation

**Figure 3:**
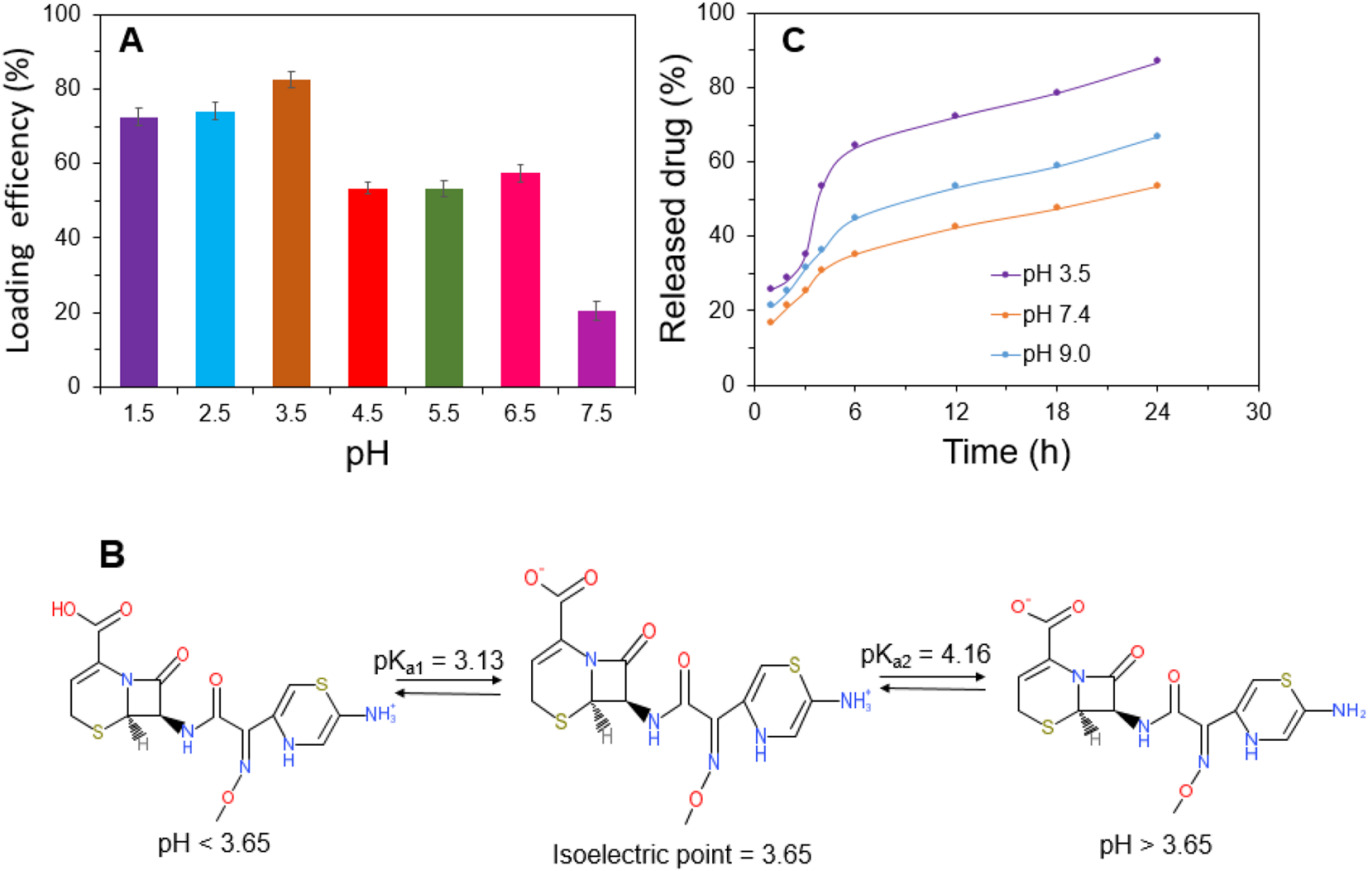
Percentage of drug loading at different pH (**A**), CFX ionic state (**B**) drug release profile of CFX under different pH at 37 ± 0.5 °C (**C**).

To investigate the effect of pH on the amount of drug loading, a series of drug encapsulation efficiency analyses were carried out under different pH at room temperature. Figure 3A shows that the percentage of drug loading at different pH and maximum efficiency was found at pH 3.5, which means the maximum amount of drug adsorbed at this pH. This fact may be confirmed by using the bipolar structure of CFX. The isoelectric point of CFX is 3.65 that means the positive part of the CFX molecule (NH_3_^+^) will be activated at pH lower than 3.65 and turn into action. The positive charge of the molecule increases with decreasing pH, as shown in Fig. 3B. The negative part of CFX (COO^−^) is activated after a rising pH greater than 3.65, and the negative charge increase with an increasing pH value of the molecule. The maximum number of positive and negative charges were found at pH 3.13 and 4.16, respectively. The probable cause of maximum adsorption found at pH 3.5 is the better interaction between the amine group of CFX and the carboxylic group of L-Cys due to a maximum number of charge differences between them. The pH value greater than 3.5, the electrostatic repulsion between CFX and L-Cys becomes higher, so the drug loading efficiency getting lower. The maximum amount of CFX loaded per grams of nanocarrier is 36 µg/ml which is briefly discussed in the supplementary information section (Fig S2).

### In vitro drug release profile analysis

To access the usability of ZnO/L-Cys as an effective nano-drug carrier, a released profile analysis of the carrier using CFX was recorded. Figure 3C shows the drug release profile from ZnO/L-Cys@CFX under different pH at 37 °C. Rapid release behavior of the carrier was found at the initial stage. Almost 30–55 percent of CFX was released in the first 4 h at different reservoir pH, but a constant release profile was achieved from 4 to 24 h. The pattern of drug release is acceptable in cases where a sufficient amount of drug is required to improve the therapeutic efficiency of antibiotics^37^. The release pattern of CFX revealed the concentration of released CFX was higher at acidic and basic concentrations than the pH of 7.4, suggesting pH-dependent drug release behavior. In acidic conditions, the drug-releasing rate was higher than that of another pH and almost 86.7% of CFX was released over 24 h releasing periods. The probable reason for this burst release behavior will be the loosening of the grafted L-Cys @CFX from ZnO bonding. The grafted composite of L-Cys@CFX became positively charged because the sulfur atom of the thiol group of L-Cys may be protonated at pH 3.5 (pKa value –SH group of L-Cys was 8.33) but the thiol group is neutral at normal blood pH^38^. So, a pH-dependent release profile of CFX from the carrier drug is found, which can be justified by previously reported work^39^.

**Figure 4:**
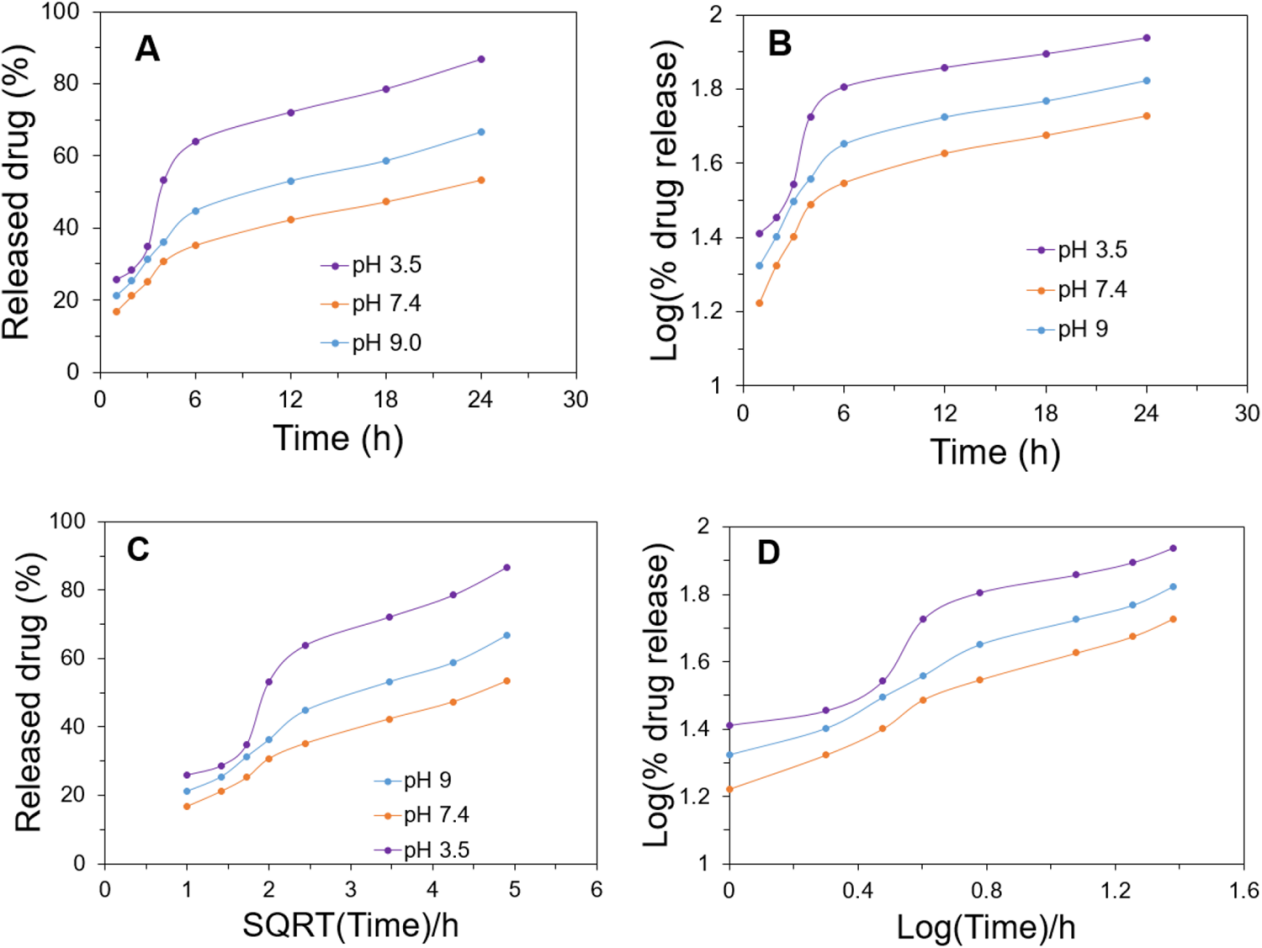
Kinetics model for release of CFX from ZnO/L-Cys@CFX (**A**) zero-order (**B**) first order (**C**) Higuchi and (**D**) Korsmeyer–Peppas model of kinetics.

To investigate the release kinetics of CFX from ZnO/L-Cys@CFX, the drug release data were fitted to a suitable model such as zero-order (percentage of drug release versus time), first-order (log percentage of drug release versus time), Korsmeyer–Peppas (log percentage of drug release versus log time), and Higuchi (percentage of drug release vs square root of time) shown in Fig. 4. To calculate the release rate constant (K) and the regression coefficient (R^2^), excel 2019 (Microsoft Corporation) was used. The best-fitted model is the Korsmeyer–Peppas model which is shown in Table 1.

**Table 1:** The release rate constant (K) and regression coefficient (R^2^) values of the different models.

Formulation	pH	Zero-order		First-order		Higuchi		Korsmeyer–Peppas	
		K_0_	R^2^	K_1_	R^2^	K_H_	R^2^	K	R^2^
ZnO/L-Cys@ CFX	9.0	1.86	0.90	0.02	0.81	11.54	0.97	0.37	0.99
	7.4	1.48	0.91	0.02	0.80	9.13	0.98	0.36	0.99
	3.5	2.54	0.82	0.02	0.72	15.53	0.91	0.41	0.93

### Antibacterial activity Of ZnO/L-Cys@CFX

#### Zone of inhibition analysis

**Figure 5:**
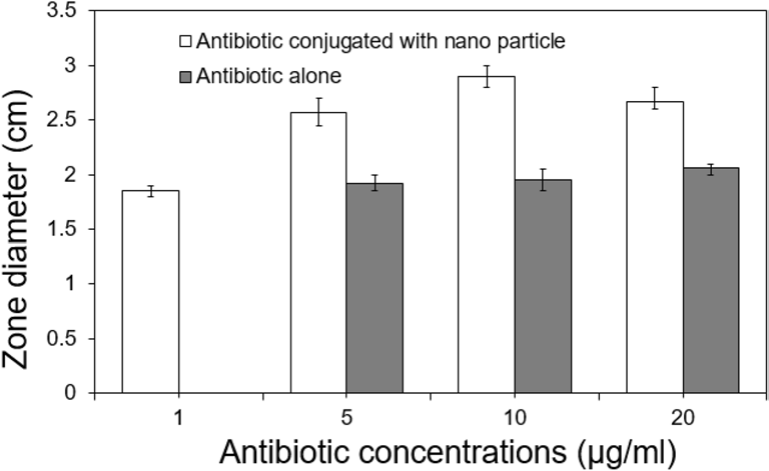
Zone diameter (cm) of *S. typhimurium* growth inhibition zone at different concentrations of CFX and CFX conjugated nanocarrier in disc diffusion method (S/N = 3).

In the disc diffusion test, we found a growth inhibition zone as low as 1.0 μg/ml ZnO/L-Cys@CFX, where no zone was found around the disc of 1.0 μg/ml ceftizoxime alone. At higher concentrations, nanoparticle conjugated antibiotics produced a more significant zone than antibiotics alone at similar concentrations (Fig. 5). The details result of zone of inhibition analysis by using disk diffusion test were interpreted in supplementary information (Table S1, Fig. S1). The nanoparticle itself did not produce any zone of bacterial inhibition. Thus, the nanoparticle conjugated antibiotic showed greater efficiency. The Ceftizoxime solution was in a non-buffered medium; when assayed using the disc agar-diffusion method, it loses potency quicker^37^. The nanoparticle conjugation might also help the antibiotic retain its potency.

#### Minimum inhibitory concentration

We found the minimum inhibitory concentration (MIC) of ceftizoxime as 20.0 μg/ml and nanoparticle conjugated ceftizoxime as 5.0 μg/ml in bacterial suspension (3.67 ± 0.47 × 10^6^ CFU/ml). We use the broth dilution method and measure the viable bacterial count by drop plate method at 6 h and 12 h of the antibiotic treatment. Surprisingly we found a higher viable count after 12 h of treatment than 6 h which can be seen in Table 2. The sensitivity of the ceftizoxime assay was reported to decrease by increasing the incubation temperature^40^. In this study, we have used the incubation temperature at 37 °C, which might also cause loss of antibiotic potency with time and thus explains the bacterial count to increase slightly with treatment. Table 3 represents different third-generation cephalosporin grouped antibiotics conjugated nanocarrier drug targets to inhibit the growth of different pathogenic bacteria. Compared to previously reported work, the ZnO and L-Cys-based nanocarrier drugs are highly effective to kill *S. typhimurium*^29,36,39–42^.

**Table 2:** Effect of nanocarrier drug compare to free drug on bacterial susceptibility (mean count ± SD) in broth dilution method.

Name	Bacterial count (CFU/ml) at 0 h	Bacterial count (CFU/ml) at 6 h	Bacterial count (CFU/ml) at 12 h
C_1_	3.67 ± 0.47 × 10^6^	2.33 ± 0.47 × 10^4^	2.5 ± 0.82 × 10^5^
C_2_	3.67 ± 0.47 × 10^6^	TFTC	TFTC
C_3_	3.67 ± 0.47 × 10^6^	TFTC	TFTC
C_4_	3.67 ± 0.47 × 10^6^	TFTC	TFTC
D_1_	3.67 ± 0.47 × 10^6^	1.67 ± 0.47 × 10^6^	1.90 ± 0.82 × 10^6^
D_2_	3.67 ± 0.47 × 10^6^	1.17 ± 0.12 × 10^6^	2.4 ± 0.82 × 10^6^
D_3_	3.67 ± 0.47 × 10^6^	5.50 ± 0.50 × 10^5^	7.50 ± 0.50 × 10^5^
D_4_	3.67 ± 0.47 × 10^6^	TFTC	TFTC
Nanocarrier	3.67 ± 0.47 × 10^6^	6.67 ± 0.94 × 10^5^	3.67 ± 0.47 × 10^5^
Control	3.67 ± 0.47 × 10^6^	TNTC	TNTC

*TNTC* too numerous to count, *TFTC* too few to count, *C*_*1*_*, C*_*2*_*, C*_*3*_*, C*_*4*_ nano carrier with 1, 5, 10, and 20 µg/ml CFX respectively, *D*_*1*_*, D*_*2*_*, D*_*3*_*, D*_*4*_ 1, 5, 10, and 20 µg/ml CFX, respectively.

**Table 3:** Comparison of antibacterial activities of some Cephalosporin grouped antibiotic-loaded nanocarrier for different targeted bacteria as previously reported.

Carrier matrix	Antibiotics	Targeted bacteria	Antibacterial activity		References
			MIC (µg/ml)	ZOI compared to the free drug (Z_a_/Z_b_)	
GO@CoFe_2_O_4_@Ag	Ciprofloxacin	*E. coli*	2.5	–	^37^
ZnO	Ciprofloxacin	*S. aureus*	20	–	^40^
AuNPs	Ciprofloxacin	*S. epidermidis*	3.9–15.62	1.05–1.09	^41^
FAuNPs	Cefotaxime	*K. pneumoniae*	0.562	–	^42^
Pectin	CFX	*B. cereus*	–	1.14	^29^
Fe3O4@BSM	Cephalexin	*S. typhimurium*	–	1.14	^43^
ZnO/L-Cys	CFX	*S. typhimurium*	5	1.5	This work

*Z*_*a*_ ZOI of nanocarrier conjugated antibiotics, *Z*_*b*_ ZOI of the reference drug.

### Effect of nanocarriers on MIC

Each component of the nanoparticle was also evaluated for the antibacterial effect in the broth dilution method. The 1.0 mg/ml ZnO nano hollow sphere, 0.5 mg/ml l-cysteine and ZnO/L-Cys nanocarrier made by nanocomposite of 1 mg/ml ZnO nano hollow sphere and 0.5 mg/ml l-cystine were tested. ZnO nano hollow sphere was found to have some bacteriostatic effect at 1.0 mg/ml and 0.2 mg/ml and not below that level which can be seen in Table 4. The major reason for this finding is the ZnO produces reactive oxygen species to damage the cell membrane mechanically^38^. Withdrawing or diluting the ZnO nano hollow sphere retain the viability of the bacterium, hence prove the non-bactericidal effect. Table 4 represents, the ZnO/L-Cys nanocarrier had some bacteriostatic effect as well but far lesser efficiency than ZnO spheres. The observation also refers that the lesser MIC and increasing zone of inhibition for nanoparticle conjugated antibiotics were not contributed by the nanoparticle, but the antibiotic itself. Nanoparticle enhances the potency or stability of the antibiotics.

**Table 4:** Comparison of antibacterial activity of different nanocarriers on bacterial growth inhibition after 6 h incubation.

Compound	Bacterial count (CFU/ml)			
Dilution (times) of nano carriers	0	5	25	125
ZnO	1.70 ± 0.16 × 10^3^	2.67 ± 0.34 × 10^3^	TNTC	TNTC
L-Cys	TNTC	TNTC	TNTC	TNTC
ZnO/L-Cys	6.03 ± 0.12 × 10^3^	4.77 ± 0.17 × 10^4^	TNTC	TNTC
Control	TNTC			

*TNTC* too numerous to count.

## Conclusion

In the present study, the ZnO nano hollow sphere was successfully synthesized from an environmentally friendly method and coated with L-Cys, confirmed by SEM, FTIR. The ZnO/L-Cys was conjugated with CFX and the resulting ZnO/L-Cys@CFX was also confirmed by the different investigations. The maximum amount of CFX conjugate with the carrier matrix was found at pH 3.5 due to electrostatic interaction between L-Cys and CFX. A pH-sensitive release profile of CFX shows around 60% of CFX released after 24 h released period at blood pH, which will be advantageous for time-consuming treatments. The growth inhibition investigation against *S. typhimurium* confirmed that the ZnO/L-Cys@CFX had been better antimicrobial properties and the ability to eradicate *S. typhimurium* more effectively than the reference drug. The MIC of CFX and ZnO/L-Cys@CFX expressed the carrier drug decrease amount CFX required to eradicate *S. typhimurium*. Therefore, ZnO/L-Cys@CFX nanocomposite could be an effective tool to restrain *S. typhimurium* type MDR pathogen.

## Supplementary Information

Supplementary Information.
