# Supplementary material for: Ceftizoxime loaded ZnO/l-cysteine based an advanced nanocarrier drug for growth inhibition of Salmonella typhimurium
Source: Sci Rep. 2021 Jul 30;11:15565. doi: 10.1038/s41598-021-95195-0 (PMC8324911; doi:10.1038/s41598-021-95195-0)
Supplement: Supplementary file 1 — Supplementary Information. [file 41598_2021_95195_MOESM1_ESM.docx]

**Supplementary information file**

**Ceftizoxime loaded ZnO/L-cysteine based an advanced nanocarrier drug for growth inhibition of *Salmonella Typhimurium***

M. S. Bacchu^1,2^, M. R. Ali^1,2^, M. A. A. Setu^3^. S. Akter^3^, M. Z. H. Khan^1,2*^

*^1^Dept. of Chemical Engineering, Jashore University of Science and technology, Jashore 7408, Bangladesh*

*^2^Laboratory of Nano-bio and Advanced Materials Engineering (NAME), Jashore University of Science and technology, Jashore 7408, Bangladesh*

*^3^Dept. of Microbiology, Jashore University of Science and technology, Jashore 7408, Bangladesh*


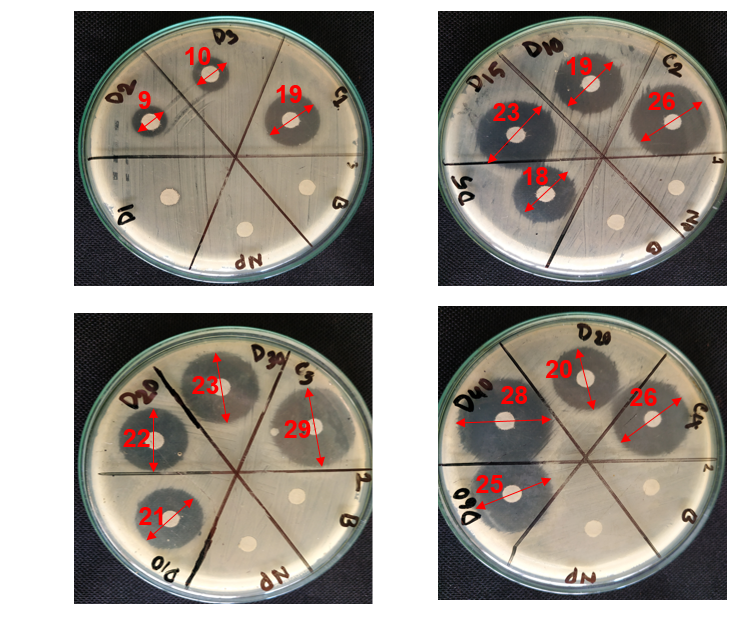
Figure S1: Comparison of the zone of growth inhibition diameter (mm) of different treatments against *S. typhimurium.*

Table S1: Zone of bacterial inhibition diameter of different antibiotics.

| Drug name | Zone Diameter (cm) | Relative standard deviation (%) (S/N=3) |
| --- | --- | --- |
| C_1_ | 1.85$\pm$ 0.04 | 2.21 |
| C_2_ | 2.57± 0.10 | 4.00 |
| C_3_ | 2.90±0.08 | 2.82 |
| C_4_ | 2.67±0.09 | 3.54 |
| D_1_ | 0 | - |
| D_2_ | 0.88±0.06 | 7.06 |
| D_3_ | 1.07±0.05 | 4.42 |
| D_5_ | 1.92±0.06 | 3.25 |
| D_10_ | 1.95±0.08 | 4.19 |
| D_15_ | 2.07±0.05 | 2.28 |
| D_20_ | 2.37±0.05 | 1.99 |
| D_30_ | 2.39±0.03 | 1.38 |
| D_40_ | 2.72±0.08 | 3.13 |
| D_60_ | 2.42±0.06 | 2.58 |
| NP | 0 | - |
| Blank | 0 | - |

C_1_, C_2_, C_3_, C_4_ – Nano carrier with 1, 5, 10, and 20 µg/ml Ceftizoxime (CFX) D_1,_ D_2,_ D_3_, D_5,_ D_10,_ D_15,_ D_20,_ D_30_, D_40,_ D_60_ – 1,2,3,5,10,15, 20, 30, 40, and 60 µg/ml CFX, NP – only nanocarrier made by nanocomposite of 1mg/ml ZnO nano hollow sphere and 0.5 mg/ml L-cystine (ZnO/L-Cys).


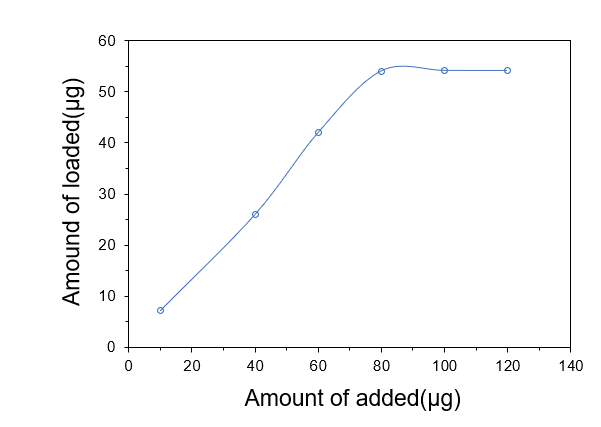


Figure S2: Amount of CFX loaded per gram of nanocarrier.

**CFX loading capacity of the nanocarrier**

The loading capacity of the nanocarrier was calculated through UV-vis Spectrometer by using the previously reported method as discussed in section 2.5. Figure S2 shows, first the values of loaded CFX increases with adding CFX but the loaded CFX amount is fixed after adding more than 80 µg/ml CFX. The highest amount of CFX loaded in the nanocarrier was approximately 54 µg/ml. So, the nanocarrier can be carried 54 µg per 1.5 g nanocarrier (36 µg/g nanocarrier).
